# Mixed infection of *Leishmania infantum* and *Leishmania braziliensis* in rodents from endemic urban area of the New World

**DOI:** 10.1186/s12917-015-0392-y

**Published:** 2015-03-20

**Authors:** Eduardo de Castro Ferreira, Israel Cruz, Carmen Cañavate, Lutiana Amaral de Melo, Agnes Antônia Sampaio Pereira, Filipe A M Madeira, Sofia Alves Nogueira Valério, Heitor Morais Cunha, Adriano Pereira Paglia, Célia Maria Ferreira Gontijo

**Affiliations:** Fiocruz Mato Grosso do Sul, Fundação Oswaldo Cruz, Campo Grande, MS Brasil; WHO Collaborating Center for Leishmaniasis, Servicio de Parasitología, Centro Nacional de Microbiología, Instituto de Salud Carlos III, Madri, Espanha; Serviço de Biologia Molecular e Bioinformática, Diretoria de Pesquisa e Desenvolvimento, Fundação Ezequiel Dias, Belo Horizonte, MG Brasil; Sete Soluções e Tecnologias Ambientais, Belo Horizonte, MG Brasil; Regional Nordeste, Secretaria de Saúde de Belo Horizonte, Belo Horizonte, MG Brasil; Departamento de Biologia Geral, Instituto de Ciências Biológicas, Universidade Federal de Minas Gerais, Belo Horizonte, MG Brasil; Grupo de Estudos em Leishmanioses, Centro de Pesquisas René Rachou, Fundação Oswaldo Cruz, Belo Horizonte, MG Brasil

**Keywords:** *Leishmania*, Hosts, Reservoir, Small mammals, Dogs, Mixed-infections

## Abstract

**Background:**

In Brazil *Leishmania braziliensis* and *L. infantum* are the principal species responsible for cutaneous and visceral leishmaniases, respectively. Domestic dogs are the main reservoirs of visceral leishmaniasis, while rodents and marsupials are the main reservoirs for cutaneous leishmaniasis. It has also been suggested that dogs could play a role in transmission of cutaneous leishmaniasis. The identification of the species of *Leishmania*, the reservoirs, and the vectors involved in each particular transmission cycle is critical for the establishment of control activities. Belo Horizonte has emerged as an endemic region for leishmaniases, however, epidemiological studies assessing the contribution of wild reservoirs to transmission are scarce in the area. The aim of this study was to investigate *Leishmania* spp. infection in possible reservoirs of an urbanized area.

**Results:**

A high rate of infection was found in small mammals (64.9%) and dogs (DG1 30.4% and DG2 48.6%). The presence of *L. infantum* and *L. braziliensis* was detected in small mammals and dogs, and mixed infections by both species were detected in rodents which, to the best of our knowledge, is the first description of this phenomenon in an urban area. Additionally, *L. amazonensis* was detected in the canine samples.

**Conclusion:**

The possible role of these animals as a source of infection of the vector of each species of *Leishmania* identified should not be overlooked and should be taken into account in future control activities. The results of mixed infection by *L. braziliensis* and *L. infantum* in cosmopolitan rodents as *M. musculus* and *R. rattus*, may have important implications in the context of the control of leishmaniasis in urban areas, especially when considering that these rodents live in close relationship with human dwellings, especially those in more precarious conditions.

## Background

The epidemiology of leishmaniases in the New World (NW) is very complex due to the participation of several species of *Leishmania*, phlebotomine vectors and mammalian hosts. NW visceral leshmaniasis (VL) is endemic or sporadic and is caused by *Leishmania* (*Leishmania*) *infantum* (syn. *L*. (*L*.) *chagasi*). Children under 10 years of age are the main affected population, but adults can also be frequently affected in foci of recent introductions. Several phylogenetically distinct species of *Leishmania* are responsible for the forms of cutaneous leishmaniasis (CL), from localized cutaneous lesions to the destructive mucosal affliction [1].

Cutaneous leishmaniasis is widespread in Brazil, which accounts for most CL cases in South America with an estimated average incidence of 100.5 cases per 100,000 inhabitants. Until the 1950s most of the cases of CL in Brazil were concentrated in the states of São Paulo, Paraná, Minas Gerais, Ceará and Pernambuco, and associated with deforestation and new human settlements. Due to the expansion of these activities the disease has spread to many other areas, including the municipality of Belo Horizonte, the capital of the state of Minas Gerais [2]. VL is also a serious public health problem in Brazil, with escalating incidence (1.85/100,000 inhabitants) and broadening geographical extension; northeastern states are particularly affected. Currently, VL is strongly linked to urban settlements in Brazil, where human made changes as well as adaptive changes in the behavior of reservoirs and vectors contribute to its prevalence. Thus, since 1980 several urban VL epidemics have taken place in Brazilian cities, including Belo Horizonte [3,4].

Several domestic, synanthropic, and wild animals have been found infected by different species of *Leishmania* in urban areas, thus the identification of sources of infection and the epidemiology of each clinical form of leishmaniasis becomes a complex matter [1,5-11]. Throughout the last decade, the detection of leishmanial DNA has been shown to be a useful approach in detecting *Leishmania* infection in dogs and other mammals in urban and sylvatic environments in Brazil, and particularly in the state of Minas Gerais [12-18].

Both CL and VL have been known to be endemic in Belo Horizonte for a long time [19,20]. This is a heavily urbanized and densely inhabited area, where transmission of leishmaniases occurs in practically all quarters of the city, resembling an urban transmission pattern already described [2,3,21,22].

In this study we aimed to use nested polymerase chain reaction (PCR) to target the *SSUrRNA* gene [23] in order to assess the presence of *Leishmania* infection in peripheral blood and different tissues obtained from dogs and small mammals from the Municipality of Belo Horizonte. The PCR method used has been validated and applied in previous studies assessing *Leishmania* infection in both humans and dogs [24,25]. In combination with DNA sequencing of positive PCR products we attempted to identify the *Leishmania* species complexes infecting the study animals. We believe that studies such as ours will contribute to an improved understanding of the epidemiology of the leishmaniases and will help to establish further control activities.

## Methods

### Study location

The study was performed from November 2007 to March 2008 in the Municipality of Belo Horizonte (capital of Minas Gerais State, Brazil). The city is built on several hills, is completely surrounded by mountains, and has several large parks in its immediate surroundings. Belo Horizonte has a global surface area of 331Km^2^, and is characterized by a subtropical climate with annual precipitation of 1491 mm and average temperature of 22°C (11°C—31°C). The human population of Belo Horizonte is 2,375,151 inhabitants [26] and is undergoing intense urbanization.

### Study design animals and samples

The number of samples was defined by estimating the proportion of the population with a confidence level of 95%, precision of 0.1 and prevalence of 50%. This value was estimated without any previous data and considering the prevalence of 50% gives the largest “n” possible. The calculated n was 100, both for dogs and for small mammals.

The project obtained permission from the Ministry of Environment Agency in Brazil, the Brazilian Institute for Environment (IBAMA), for capture and euthanasia of small mammals.

The points of collecting samples of dogs and small mammals in domiciliary and peridomestic areas were chosen considering the presence of human cases of VL and/or LT in the household, or close to it, in the three years preceding the start of research.

The study was based on a sample of 189 animals originating from two sources: i) 97 small mammals (62 rodents and 35 marsupials) captured with Tomahawk traps from peri-domestic habitats (20 houses, each one with two traps) and from forested areas in early succession around different households in Belo Horizonte (three tracks, each containing 15 traps separated by a distance of about 20 meters) (Table 1). The sampling effort, at the end of the six campaigns, was 1200 traps in peridomestic area and 1350 traps in the forested area, adding up to a total of 2550 traps during the period of study ; and ii) 92 domestic dogs (with owners) from the same households from which small mammals were captured (hereinafter dog group-1, DG1).

**Table 1:** **Number of individuals of small mammal species captured in each habitat between June 2006 to November 2007 in Belo Horizonte, Minas Gerais, Brasil**

**Order**	**Species**	**Forest**	**Peridomestic**	**Total**
Rodentia	*Mus musculus*	4	20	24
	*Rattus rattus*	8	11	19
	*Rattus norvegicus*	1	8	9
	Necromys lasiurus*	5	0	5
	Cerradomys subflavus*	5	0	5
Didelphimorphia	Didelphis albiventris*	31	3	34
	Didelphis aurita*	1	0	1
	Total	55	42	97

*Native species.

An additional set of dog samples was included in this study (hereinafter dog group-2, DG2). This group was made up of DNA extracted from peripheral blood, bone marrow and ear skin of 70 dogs obtained in study location during a previous survey [27] , to evaluate whether there were changes in the profile of infection in dogs from the same area, in both periods studied, applying the same methodology on samples of the two groups.

Small mammals were anaesthetized and sacrificed, and then samples from tail skin, ear skin, liver, spleen, and bone marrow were collected. Peripheral blood samples were taken from dogs. Peripheral blood and bone marrow samples were preserved in EDTA tubes at −20°C whereas the other tissue samples were stored in 70% ethanol at −20°C until analysis.

Informed consent was obtained from each dog owner before sampling. The procedures employed for the collection of clinical material from dogs and small mammals were carried out according to the Ethical Principles in Animal Experimentation and the project was licensed by the Ethics Commission on the Use of Animals/FIOCRUZ under reference number P0119-02.

### DNA extraction

The *Genomic Prep Cells and Tissues DNA Isolation Kit*® (GE Healthcare) was used for DNA extraction from skin, liver, and spleen samples and the *Column Chromatography*-*GFX Genomic DNA Blood Purification System*® (GE Healthcare) was used for peripheral blood and bone marrow samples. In both cases the instructions provided by the manufacturer were followed.

### Detection of *Leishmania* DNA

Detection of *Leishmania* DNA was done by means of *Leishmania* nested-PCR (LnPCR) targeting the *SSUrRNA* gene, as described elsewhere [24]. This protocol is specific to the genus *Leishmania* and uses the primer pair R221 (5’-GGT TCCTTT CCT GAT TTA CG-3’) and R332 (5’-GGC CGGTAA AGG CCG AAT AG-3’) in the first reaction, and R223 (5’-TCC CAT CGC AAC CTC GGT T-3’) and R333 (5’-AAA GCG GGC GCG GTG CTG-3’) in the nested reaction. The PCR reagents (Biotools B&M Labs, SA) and the thermal cycler (GenAmp PCR System 9800, Applied Biosystems) were used following the conditions and the cycling program previously described [24]. Every assay included DNA extraction negative and positive controls and PCR negative and positive controls. The presence of the final specific positive LnPCR product (353 bp) was assessed by 1.5% agarose gel electrophoresis, ethidium bromide staining and UV visualization. The detection limit observed was equivalent to 1 and 10 parasites/uL in culture samples of reference strains of *L. infantum* and *L. braziliensis*, respectively.

### DNA sequencing

For species identification, all LnPCR positive products were excised from the gel and purified using the *QIAquick Gel Extraction Kit* (QIAGEN) following the protocol provided by the manufacturer. Direct sequencing of the purified LnPCR positive products was performed with forward and reverse primers using the *Big*-*Dye Terminator Cycle Sequencing Ready Reaction Kit V3.1* and the automated ABI PRISM 377 DNA sequencer (Applied Biosystems). Sequences thus obtained were analyzed and edited using Lasergene® sequence analysis software (DNASTAR). The edited sequences were compared with those deposited at GenBank.

### Statistical analysis

The differences of positive frequencies were analyzed by chi-square test (x^2^) and for multiple comparisons was used the Bonferroni method. The level of significance was 5%.

## Results

We captured a total of five species of rodents and two species of marsupials in both habitats (Table 1). Native wild species were captured mostly in forested areas while in the peri-domestic areas all species captured, except the white-eared opossum (*Didelphis albiventris*), were exotic and synanthropic.

**Figure 1:**
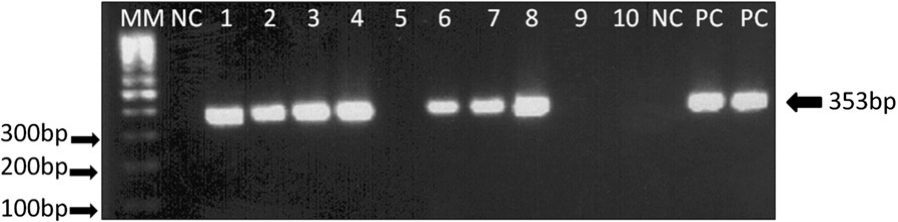
**Agarose gel electrophosis of the amplication products from LnPCR for the SSUrRNA gene.** Samples: MM:100 bpmolecular size marker; NC: Negative Control; 01 to 10: amplifield DNA from mammal samples; PC: Positive control-*L.braziliensis* (MOHM/BR/75/M2903)DNA.

After LnPCR analysis of the different biological samples any animal presenting an LnPCR positive result (Figure 1) in at least one of the biological samples tested was considered infected. Small mammals (N = 97) had the highest infection rate (47.4--83.3%), which varied according to species (details in Table 2). Among dogs, DG1 (N = 92) showed an infection rate of 30.4%, while DG-2 (N = 70) had an infection rate of 48.6% (Table 2). Among small mammals, samples from liver, spleen and tail skin showed a higher infection rate (22.4--33.7%) than samples taken from ear skin and bone marrow (5.2--10.2%). No significant differences were observed in the infection rate of the different samples taken from DG2 (Table 3).

**Table 2:** **Positivity of LnPCR in samples of rodents, marsupials and dogs from Belo Horizonte, Minas Gerais, Brazil**

**Host specie**	**Sample**	**LnPCR**
*M. musculus*	**	83.3%
*R. rattus*	**	47.4%
*R. norvegicus*	**	66.7%
*N. lasiurus*	**	60%
*C. subflavus*	**	80%
*D. albiventris*	**	61.8%
*D. aurita*	**	0%
*C. familiaris* DG1	Blood	30.4%
*C. familiaris* DG2	**	48.6%

**Positivity in at least one evaluated tissue, including blood, ear skin, and bone marrow for *C. familiaris* (2) and ear skin, tail skin, liver, spleen, and bone marrow for the other hosts.

**Table 3:** Positivity of *Leishmania sp.* specific PCRs performed on DNA extracted from different tissues of small mammals and dogs

**Host specie**	**Ear skin**	**Tail skin**	**Bone marrow**	**Liver**	**Spleen**	**Blood**
*M. musculus*	12.5%	17.4%	5%	56.5%	60.9%	NR
*R. rattus*	15.8%	5.3%	5.3%	26.3%	5.3%	NR
*R. norvegicus*	33.3%	33.3%	0%	55.5%	0%	NR
*N. lasiurus*	0%	0%	0%	60%	60%	NR
*C. subflavus*	0%	0%	20%	60%	40%	NR
*D. albiventris*	3%	38.2%	6.1%	14.7%	20.6%	NR
*D. aurita*	0%	0%	0%	0%	0%	NR
*C. familiaris* DG1	NR	NR	NR	NR	NR	31.5%
*C. familiaris* DG2	25.7%	NR	32.9%	NR	NR	21.4%

NR_Unrealised.

DNA sequence analysis of the amplified fragment of the *SSUrRNA* gene indicated that species from the *L. brazilensis* complex were infecting 54%, 82.1% and 5.9% of the LnPCR positive small mammals, DG-1 and DG-2, respectively. Infection by species of the *L. donovani* complex was determined in 12.7%, 17.9% and 82.3%, of the LnPCR positive small mammals, DG1 and DG2, respectively. When different *Leishmania* species complexes were identified in different biological samples from the same animal the infection was considered mixed; mixed infection by species of the *L. donovani* complex and *L. braziliensis* complex were detected in 4.7% of the LnPCR positive small mammals (two *Mus musculus*, and one *Rattus rattus*). Infection by *L. amazonensis* was observed in 5.9% of the LnPCR positive dogs of DG2. Due to weak amplification it was not possible to obtain accurate DNA sequences for species complex identification in 28.6% and 5.9% of the LnPCR positive small mammals and dogs of DG2, respectively. Details on the species complex identification for each animal species are provided in Table 4.

**Table 4:** **Identification of** ***Leishmania*** **by the sequencing of the LnPCR product in samples of small mammals and dogs**

**Host species (n)**	**Sample**	**Leishmania Complex**				
		***L*** **.** ***b***	***L*** **.** ***d***	***L*** **.** ***b*** **+** ***L*** **.** ***d***	***L*** **.** ***m***	***Leishmania sp***
*M. musculus* (20)	**	55%	10%	10%	0%	25%
*R. rattus* (09)	**	44.4%	0%	11.2%	0%	44.4%
*R. norvegicus* (06)	**	66.6%	16.7%	0%	0%	16.7%
*N. lasiurus* (03)	**	100%	0%	0%	0%	0%
*C. subflavus* (04)	**	50%	25%	0%	0%	25%
*D. albiventris* (21)	**	47.6%	19%	0%	0%	33.4%
*C. familiaris* DG1 (28)	Blood	82%	18%	0%	0%	0%
*C. familiaris* DG2 (34)	**	5.9%	82.3%	0%	5.9%	5.9%

**Identification carried out in at least one positive tissue by the PCR, including ear skin, tail skin, liver, spleen, and bone marrow for the small mammals and blood, ear skin, and bone marrow for DG2. *L. b* (*Leishmania braziliensis*), *L. d* (*L. donovani*), *L.m* (*L.mexicana*).

## Discussion

The present study represents the first time that PCR targeting the *SSUrRNA* gene and further DNA sequencing has been used in an epidemiological study on samples from both domestic dogs and small mammals. Furthermore, this study adds to the previous work by Marcelino and colleagues [28], who applied the same methodology to confirm infection by the *L. braziliensis* species complex in a sample of *Rattus norvegicus*. This methodology, however, only allows identification to the level of species complex and further analysis using other DNA targets, such as the *Hsp70* gene, would be necessary to allow identification to the species level [29].

The present study also identified, for the first time, mixed infection by the *L. donovani* species complex and the *L. braziliensis* species complex in three small mammals (two *M. musculus* and one *R. rattus*) captured in peri-domestic habitats of three different households of Belo Horizonte. The overlapping of transmission cycles of different species of *Leishmania* in a given area can lead to the occurrence of mixed infections, as has been observed by other authors [30-33]. This is an astonishing finding since these two species of rodents are commonly found in urban areas close to domestic habitats and in association with inadequate hygiene and environmental disorganization.

In epidemiological studies in areas of occurrence of different species of *Leishmania*, the collection of biological samples from different tissues increases not only the probability of detecting mixed infections [30], but also the chance of detecting the parasite itself, as shown in Table 3.

Another fact that caught our attention was the presence of domestic dogs infected by *Leishmania* belonging to the *L. mexicana* complex. This finding, indicates that sympatric transmission of *L. amazonensis* species of the *L. braziliensis* and *L. donovani* complexes is occurring.

The species of *Leishmania* most prevalent in small mammals, both in the forested and peri-domestic areas, was *L. braziliensis* followed by *L. infantum*. Although none of the small mammals were found to be infected by *L. amazonensis*, the possibility should not be entirely ruled out since it was not possible to identify the etiologic agent of 29.7% of the small mammals that were infected. This is especially true if we consider that this species of *Leishmania* is encountered in dogs [34], and other authors have reported the detection of infection by this species in small mammals from different regions of Brazil [14,35,36].

The identification of the parasites present in infected dogs and small mammals has revealed species belonging to the *Leishmania braziliensis*, *Leishmania mexicana*, and *Leishmania donovani* complexes. This, combined with the results of Saraiva and colleagues [37] who detected the presence of these species in phlebotomines, confirms the occurrence of an active transmission cycle of these different species in Belo Horizonte.

In captures of sandflies taken during the same period of DG2 samples collection, 68% of specimens were *L. longipalpis* [38]. Moreover, in a survey conducted in the same area and period that the DG1 and small mammal samples were collected, 75% of the sandfly specimens captured were *L. whitmani* [39]. The above mentioned, prevalence of *L. infantum* in DG2 and *L. braziliensis* in DG1 and small mammals are understandable given the predominance of the respective vectors in the different periods.

Most of the necessary conditions for incriminating a species as a reservoir of *Leishmania* [11], was observed in the dogs and small mammals sampled in our study.

Among commensal synanthropic species, the rat (*R. norvegicus*), the roof rat (*R. rattus*), and the mouse (*M. musculus*) are particularly important because they have a cosmopolitan distribution and are responsible for most of the economic losses and health caused to man [40]. The high rates of infection detected in these species, as well as the possibility of harboring different species of *Leishmania*, coupled with the proliferation of these animals in urban areas suggest the possibility that they are contributing to the maintenance of the transmission cycles of *Leishmania* sp. Moreover, these synanthropic species were also trapped in the forested areas. Freitas and colleagues [41] also suggest that *M. musculus* participates in the transmission cycle of *L. braziliensis* in an endemic area of the state of Mato Grosso, Brazil.

With respect to the white-eared opossum *D. albiventris*, this species showed a high rate of infection in tail skin (38.2%) with parasites characterized as *L. braziliensis* or *L. infantum*, which corroborates the findings of numerous authors [5,16,18,36,42,43]. Although most specimens were captured in the forested area, *D. albiventris* has a large home range and highly synanthropic habits, which could make them a link between wild and urban transmission cycles.

The presence of dogs infected with *L. braziliensis* and *L. infantum* in a city like Belo Horizonte is consistent with what has been described by other authors in LT and LV endemic areas [3,44,45]. In addition, the high percentage of dogs infected by *L. braziliensis* in an area with LV transmission should be evaluated by other methods, besides the serological diagnosis, to perform euthanasia of seropositive dogs, as recommended by the Ministry of Health of Brazil for LV control [46]; it is known the possibility of cross-reactions by serological tests used in Brazil for diagnosis of dogs infected by *L. braziliensis* [27].

The present study shows that three different leishmanial entities (*L. donovani* species complex, *L. braziliensis* species complex, and *L. mexicana* species complex) are present as etiological agents in the infection of domestic dogs from the Belo Horizonte, which is considered an endemic area for VL and CL. Additionally, small mammals in peri-domestic and forested areas are also infected by parasites of the *L. donovani* species complex or the *L. braziliensis* species complex.

## Conclusions

Mixed infection by *L. braziliensis* and *L. infantum* in cosmopolitan rodents as *M. musculus* and *R. rattus*, may have important implications in the context of the control of leishmaniasis in urban areas, especially when considering that these rodents live in close relationship with human dwellings, especially those in more precarious conditions. The possible role of these animals as a source of infection for the vector of each *Leishmania* sp. identified in Belo Horizonte should not be overlooked and should be taken into account in future control activities. Furthermore, studies of the hosts of *Leishmania* spp., the potential reservoirs of these parasites, should be encouraged in several endemic areas of the disease in order to understand the real role of the different host species in the complex eco-epidemiology of leishmaniasis.

## Abbreviations

NW: New World
*L*.: *Leishmania*
VL: Visceral leishmaniasis
CL: Cutaneous leishmaniasis
PCR: Polymerase chain reaction
DG1: Dog group 1
DG2: Dog group 2
LnPCR: *Leishmania* nested PCR
